# Long-Term Follow-Up of a Patient with McCune–Albright Syndrome: A Case Report

**DOI:** 10.3390/jcm14176101

**Published:** 2025-08-28

**Authors:** Yuto Shoji, Satoru Kusaka, Kana Kawashima, Shuma Hamaguchi, Meiko Tachikake, Tatsuya Akitomo, Ryota Nomura

**Affiliations:** 1 Department of Pediatric Dentistry, Graduate School of Biomedical and Health Sciences, Hiroshima University, Hiroshima 734-8553, Japan; shouji12@hiroshima-u.ac.jp (Y.S.); kanana25@hiroshima-u.ac.jp (K.K.); syuumai@hiroshima-u.ac.jp (S.H.); meikosan@hiroshima-u.ac.jp (M.T.); rnomura@hiroshima-u.ac.jp (R.N.); 2 Department of Pediatric Dentistry, Hiroshima University Hospital, Hiroshima 734-8551, Japan; higechi@hiroshima-u.ac.jp

**Keywords:** bisphosphonates, case report, fibrous dysplasia, McCune–Albright syndrome

## Abstract

**Background/Objectives:** McCune–Albright syndrome (MAS) is a rare disease characterized by the triad of fibrous dysplasia (FD), café au lait skin macules, and hyperfunctioning endocrinopathies. Although there are many case reports of MAS, few have discussed long-term oral management. We describe the long-term follow-up of an MAS patient over 15 years. **Case Presentation:** A male patient aged 13 years and 7 months was referred to our department with a chief complaint of difficulty with toothbrushing. He was diagnosed with MAS at 9 years, and bisphosphonate therapy was started. We continued to review the patient periodically and extracted several primary teeth with no adverse effects such as the medication-related osteonecrosis of the jaw (MRONJ). We evaluated the changes in FD using facial photographs, and facial asymmetry worsened over time until the age of 19, when surgery was performed. Although improvement was observed after surgery, there was a tendency for recurrence up to the age of 25 years. **Conclusions:** Continuous dental support over 15 years has prevented oral disease and minimized the need for surgical procedures such as tooth extractions, which are factors in MRONJ. The worsening of FD on the left side caused facial asymmetry until the age of 25 years; however, the asymmetry may have stabilized with the development of FD on the right side and with age-related changes. It is important for dental professionals to provide MAS patients with appropriate oral health instruction and oral management, taking changes in FD into consideration.

## 1. Introduction

McCune–Albright syndrome (MAS) is a rare genetic disorder associated with activating mutations in the guanine nucleotide-binding protein, alpha-stimulatory (GNAS) gene that affects multiple organs and was first described by McCune and Albright in 1937 [1]. MAS is defined by the classic triad of fibrous dysplasia (FD) of bone, café au lait skin macules, and hyperfunctioning endocrinopathies. Hyperfunctioning endocrinopathies include gonadotropin-independent precocious puberty, growth hormone excess, non-autoimmune hyperthyroidism, hyperprolactinemia, and neonatal hypercortisolism [2,3]. A diagnosis of MAS requires the presence of at least two of the above clinical features [3]. MAS is a rare disease, with an estimated prevalence of approximately 1/100,000 to 1/1,000,000 [4].

The clinical manifestations of MAS vary greatly, with the most typical being FD, which is a skeletal disorder resulting in deformity, fractures, pain, and functional impairment [5,6]. It involves any part or combination of the craniofacial, axial, or appendicular skeleton, and can range from an isolated, asymptomatic monostotic lesion discovered incidentally to severe, disabling polyostotic disease [7]. Most MAS patients (90%) have FD lesions in the craniofacial area, and maxillomandibular FD is also associated with dental developmental disorders, malocclusion, and a high caries index [8].

Although there are many case reports of MAS, most of them discuss clinical features or diagnosis, and few describe long-term changes in FD [3,9,10,11]. We encountered a patient who was diagnosed with MAS and presented with facial asymmetry and malocclusion due to FD since childhood. This report describes the oral management of this MAS patient and analyzes facial asymmetry using objective indices to clarify changes in FD by age.

## 2. Case Presentation

**Figure 1:**
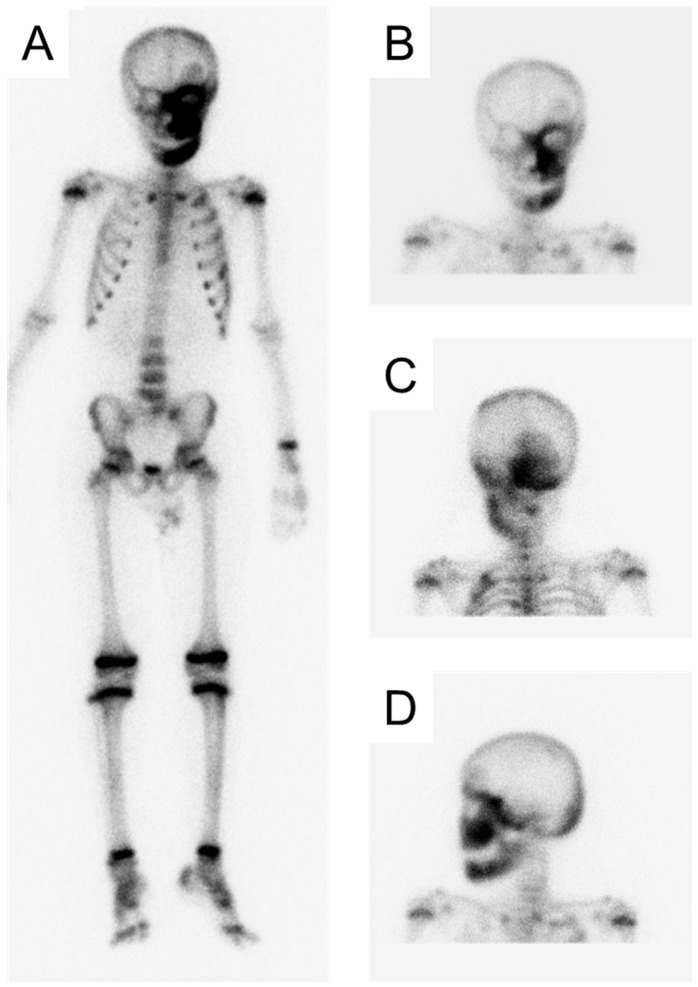
Bone scintigraphy at the age of 9 years and 0 months ((**A**): Whole body, (**B**): Anterior head, (**C**): Posterior head, (**D**): Left-lateral head).

A boy aged 13 years and 7 months who had difficulty with toothbrushing was referred to our department from the oral surgery department in our hospital. Swelling in the left cheek region had been present since the age of 5 and he had presented at the oral surgery department at the age of 9 years. He was diagnosed as precocious puberty, and a biopsy of the maxillary also revealed FD, confirming the diagnosis of MAS. Additionally, bone scintigraphy revealed an abnormal accumulation of the radioisotope 99mTc-HMDP in the left facial bones (frontal bone, maxilla, mandible), temporal bone, sphenoid bone, occipital bone, ribs, thoracic vertebrae, and left sacrum (Figure 1). Bisphosphonate therapy (oral alendronic acid, 5 mg/day) was commenced to prevent fractures. At the age of 11, the patient’s left eyesight was deteriorating due to the enlargement of the cephalic FD, and optic canal decompression surgery was performed.

**Figure 2:**
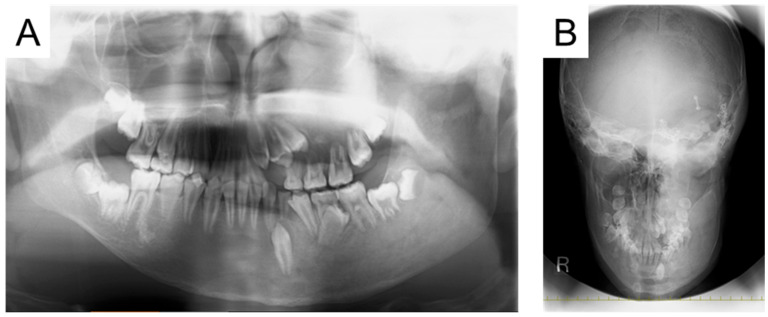
Radiographic examination at the age of 12 years and 9 months ((**A**): Panoramic photograph, (**B**): Antero-posterior-view skull).

At the first visit to our department, swelling was observed in the left cheek, and the face was asymmetrical. The left maxillary and mandibular bodies were swollen, and a crossbite was observed in the left molar region. Radiographic examinations taken in oral surgery at the age of 12 years and 9 months are shown in Figure 2. Multiple primary teeth were retained, and ground-glass opacity was detected in the left maxillary and mandibular bone. The initial examination detected no dental caries, and we continued with oral management to prevent oral disease.

**Figure 3:**
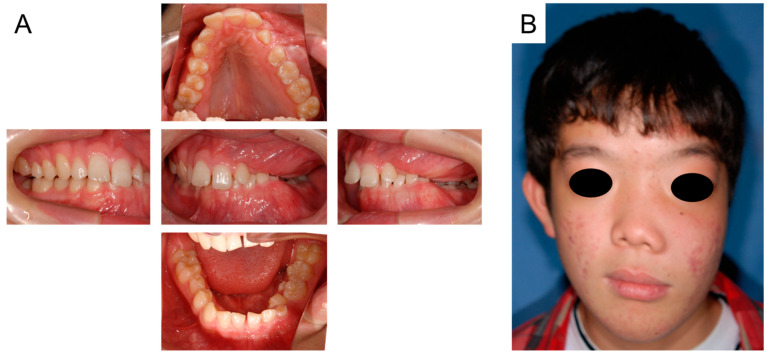
Oral and facial photographs at the age of 14 years and 9 months ((**A**): Intraoral photographs, (**B**): Facial photograph).

Oral and facial photographs at the age of 14 years and 9 months are shown in Figure 3A,B. The right maxillary second premolar and left maxillary canine erupted naturally in the oral cavity. Although the retention of the right mandibular primary second molar was prolonged, we continued to simply observe it because of the risk of medication-related osteonecrosis of the jaw (MRONJ). Facial asymmetry was noted due to FD; however, there were no other pathological findings.

**Figure 4:**
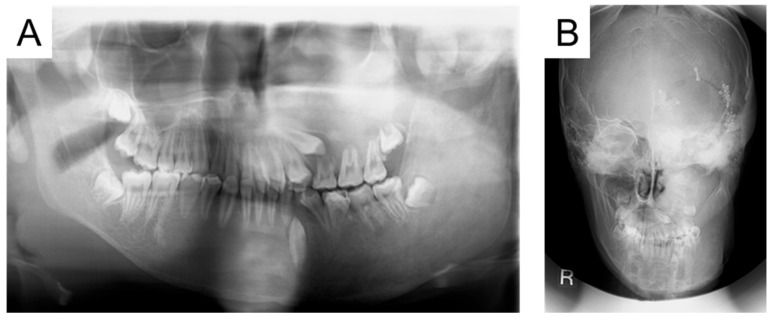
Radiographic examination at the age of 15 years and 6 months ((**A**): Panoramic photograph, (**B**): Antero-posterior-view skull).

At the age of 15 years and 6 months, the left maxillary first premolar had partially erupted, and a crown fracture was detected in the primary first molar (Figure 4). We considered that the tooth would not fall out naturally and required extraction. We explained the risk of MRONJ and extracted the tooth that day, but no adverse reactions occurred.

**Figure 5:**
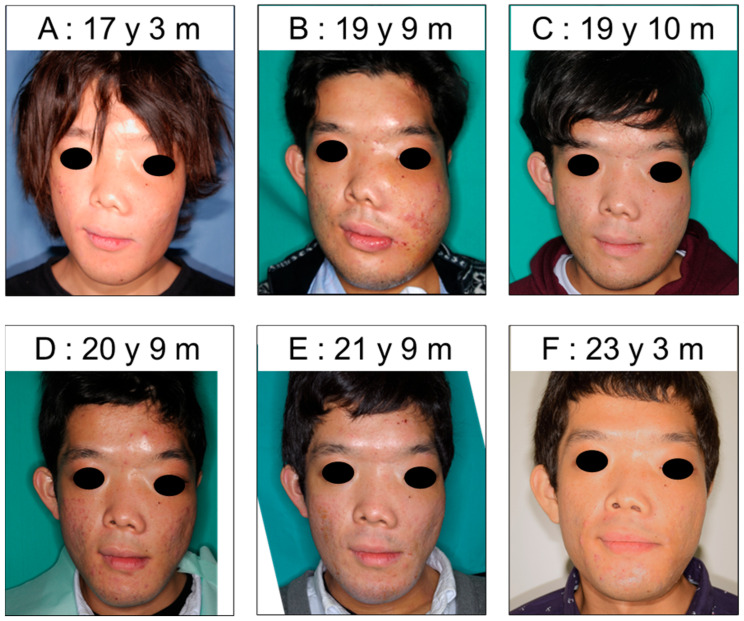
Facial photographs ((**A**): 17 years and 3 months, (**B**): 19 years and 9 months, (**C**): 19 years and 10 months, (**D**): 20 years and 9 months, (**E**): 21 years and 9 months, (**F**): 23 years and 3 months).

Bisphosphonate therapy was discontinued at the age of 19 years and 5 months. Over time, the FD had progressed, and the facial asymmetry had worsened (Figure 5A,B). At the age of 19 years and 9 months, maxillary and mandibular osteotomies were performed under general anesthesia, and the facial asymmetry improved. We observed the changes in facial appearance at follow-up appointments and detected a slight increase in FD with increasing age (Figure 5C–F).

**Figure 6:**
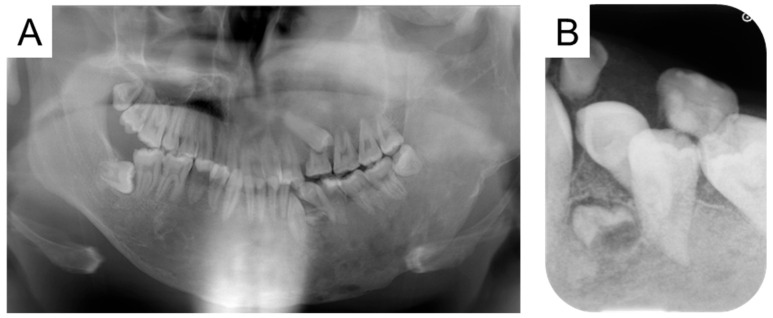
Radiographic examination at the age of 23 years and 11 months ((**A**): Panoramic photograph, (**B**): Periapical photograph).

**Figure 7:**
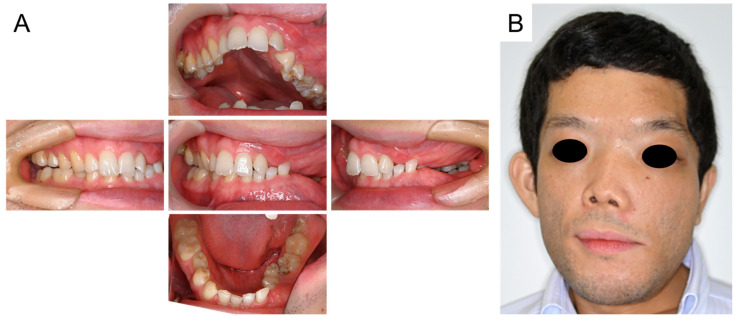
Oral and facial photographs at the age of 27 years and 4 months ((**A**): Intraoral photographs, (**B**): Facial photograph).

**Figure 8:**
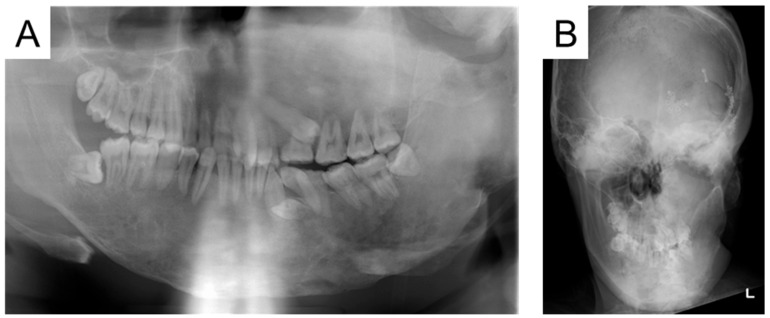
Radiographic examination at the age of 27 years and 10 months ((**A**): Panoramic photo-graph, (**B**): Antero-posterior-view skull).

We detected the supernumerary tooth in the mandibular left canine region at the age of 23 years and 11 months and continued to follow up (Figure 6). The fractured left mandibular primary second molar was extracted in the oral surgery department at the age of 24 years and 10 months with no postoperative problems. Intraoral and facial photographs at the age of 27 years and 4 months are shown in Figure 7. The left maxillary primary second molar and the left mandibular primary canine were retained. Although several teeth had exogenous stains, none were carious. Additionally, there was no occlusal contact in the right molar region. At the age of 27 years and 10 months, no obvious changes were otherwise remarkable, except for the growth of supernumerary tooth (Figure 8).

**Figure 9:**
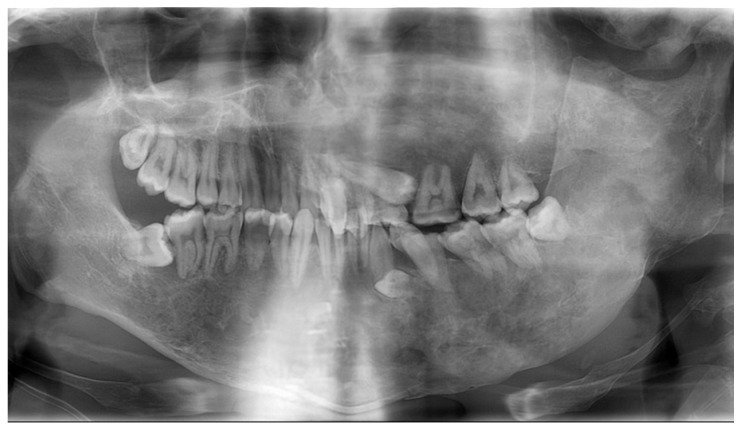
Panoramic radiograph at the age of 29 years and 10 months.

**Figure 10:**
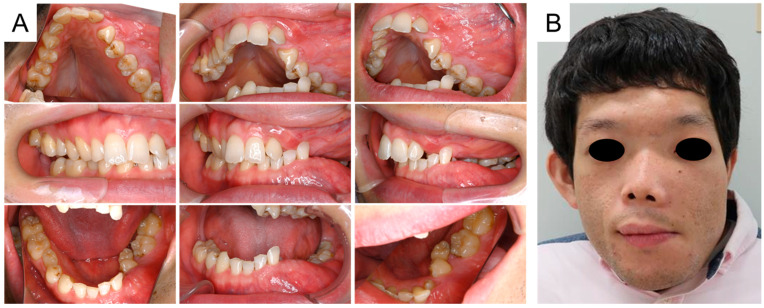
Oral and facial photographs at the age of 30 years and 5 months. ((**A**): Intraoral photographs, (**B**): Facial photograph).

A panoramic radiograph at 29 years and 10 months indicated that the ground-glass opacity had extended beyond the midline into the right molar region (Figure 9). Dental caries treatment on the distal surface of the left maxillary first molar was performed at that point. It was decided to continue monitoring the patient’s malocclusion in accordance with the patient’s wishes, as there was a possibility that FD would cause changes in the future. At the age of 30 years and 3 months, the left mandibular primary canine was extracted by the oral surgery department to facilitate eruption of the permanent canine. Although the patient is now over 30 years old, the left maxillary primary second molar is still retained (Figure 10), and the left maxillary second premolar has not erupted. There are many impacted teeth such as supernumerary teeth or third molars left, and the distal surface of the right mandibular second molar has a risk of bone resorption. Considering the risk of MRONJ, additional surgery is not advisable. We continue to check the progress of the impacted tooth or FD and provide oral care at our hospital. To date, no evidence of MRONJ has been found.

**Figure 11:**
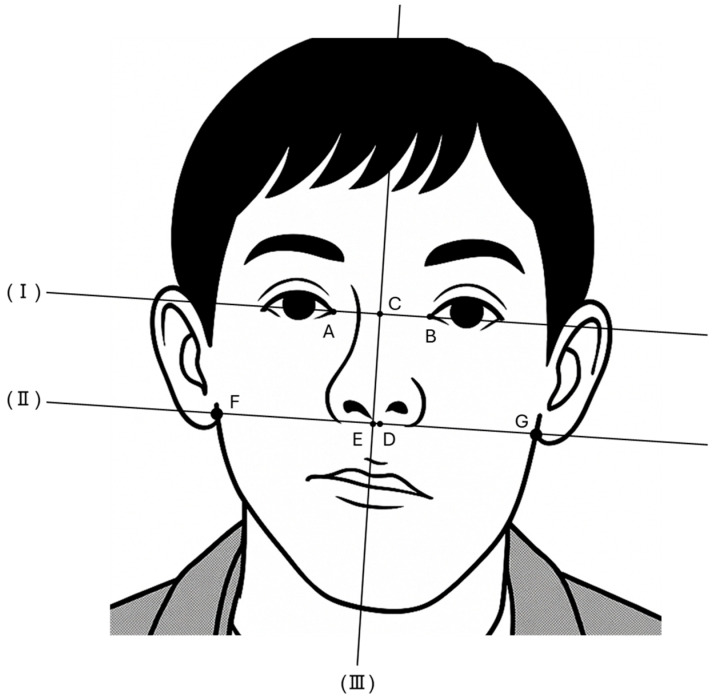
Landmark and reference lines used in the study [12].

We evaluated changes in FD over time using secular facial photographs. Kato et al. (2009) devised an objective index to evaluate facial asymmetry using facial photographs [12]. In this study, we modified the measuring items, and evaluations were modified from the previous report [12]. Details of the measurement items and reference lines used in this study are shown in Table 1 and Figure 11, and definitions of the asymmetry ratios are shown in Table 2.

The changes in FD are shown in Table 3. The asymmetry ratios from both the midline and the subnasale increased with age, reaching a maximum at 19 years and 9 months of age, when maxillary and mandibular osteotomies were performed. Osteotomies significantly improved the facial asymmetry, resulting in an asymmetry ratio from the midline of 0.78. At the age of 21 years and 9 months, both asymmetry ratios had decreased; however, they had worsened again by the age of 25 years and 1 month. Five years have passed since then, and the asymmetry has shown a slight tendency to improve.

## 3. Discussion

Most MAS patients have FD in the craniofacial region; therefore, long-term follow-up is important. We provided a follow-up of an MAS patient over 15 years and investigated changes in FD over time using facial photographs. To our knowledge, this is the first report of the long-term dental follow-up of an MAS patient including changes in FD. The timeline of the patient is shown in Table 4.

At the first visit, the patient was 13 years and 7 months old; however, four primary teeth were retained. Although there is no known association with FD, all the retained teeth were on the left side where the FD occurred. The patient had received bisphosphonate therapy since the age of 9 years, and it has been reported that eruption disturbances occur in patients who receive bisphosphonate treatment during childhood [13]. Additional surveys are needed to investigate prolonged retention of primary teeth in MAS patients.

Bisphosphonates are used to prevent recurrent fractures, and they act as antiresorptive agents [14]. On the other hand, bisphosphonate treatment increases the risk of MRONJ, so invasive dental procedures should be avoided whenever possible [15,16]. We were aware of the risk of MRONJ and continued the follow-up of the patient. Although bisphosphonate therapy continued until the age of 19 years and 5 months, a primary tooth was extracted during the medication period because of crown fracture. Fortunately, no adverse events occurred. Although many cases of MRONJ have been reported in adults, no study has reported childhood MRONJ [17]. Mitsuhata et al. (2022) investigated the extraction of primary teeth in 15 children with a history of bisphosphonate administration, and no adverse events were reported [17]. Minimally invasive extractions such as those in primary teeth with resorbed roots may carry a lower risk of MRONJ. However, susceptibility to MRONJ may be increased in an adult who has used bisphosphonate therapy continuously for a long period of time from childhood because of the reduced bone turnover [17,18]. Although MRONJ did not occur in the present case, these previous reports highlight the importance of long-term oral management in MAS patients who received bisphosphonate therapy.

We evaluated the changes in FD using serial facial photographs at different ages. We compared the results based on both eyes and the nose. The nasal bridge was deviated to the right because of FD; therefore, the asymmetry ratio from the subnasale was higher than that based on the midline of both eyes at all points. The asymmetry ratio worsened from the age of 14 to 19 years, and maxillary and mandibular osteotomies were performed when the patient was 19 years and 9 months. The surgery dramatically improved the asymmetry, and the scores for both indicators improved by nearly 15%. Facial asymmetry tended to improve for approximately 2 years after surgery. However, at the age of 25, it had worsened again to some extent, indicating a recurrence of FD. Between the ages of 27 and 30, the ratios showed a slight downward trend, and the asymmetry improved. A panoramic radiograph at the age of 29 years and 10 months revealed that the FD had extended beyond the midline to the right side. Additionally, soft tissues in different areas of the face become selectively thickened or thinned with aging [19]. The decline in the scores after the age of 27 years may suggest that the improved asymmetry was not due to a decrease in FD, but to the spread to the right side and other factors such as aging. Additional investigation is needed to clarify this supposition. The asymmetry peaked at the age of 19 years before the osteotomy, and then recurred at the age of 25 years in the present case. An additional survey with a larger sample size is needed to determine whether FD increases with age throughout life.

As mentioned above, the characteristics of MAS include FD, café au lait skin macules, and hyperfunctioning endocrinopathies [2,3]. MAS patients sometimes also have pigmentation in the oral cavities [20]. Pichard et al. (2014) reported 4 MAS patients who developed oral mucosal pigmentation during childhood or early adulthood, and mucosal pigmentation in MAS develops later in life (ages 7–20 years) [20]. In the present case, the patient was diagnosed with MAS because of precocious puberty and FD. We followed the patient’s long-term course from age 13 to 30 years; however, no occurrence of oral mucosal pigmentation was detected.

Akintoye et al. (2003) investigated the dental characteristics of 32 patients with FD, and eighty-four percent had FD in the maxilla and/or mandible [21]. In addition, malocclusion was the most common abnormality, which was present in 81% of patients [21]. In the present case, the patient is currently 30 years old and has a retained left maxillary primary second molar, with an impacted successor permanent tooth. In addition, supernumerary tooth was also impacted in the left mandibular canine region. The teeth are also misaligned because of FD; however, the patient wishes to remain under observation rather than undergo further treatment. Although some teeth have been restored, no root canal treatment or extractions have been undertaken. Appropriate oral health instruction has been provided to the patient regarding the malocclusion, and continual dental check-ups for over 15 years have helped to improve the patient’s quality of life. This report highlights the importance of regular dental check-ups.

This report has some limitations. First, because this is a patient report spanning 15 years, there were periods when intraoral photographs from the initial visit were not available, and medical records such as the pathohistological image of the biopsy were incomplete. Additionally, although the photographs were always taken from the front, they were not standardized. Further investigation is needed to clarify the changes in FD over time using standardized facial photographs in the future.

## 4. Conclusions

We described the oral management of an MAS patient over 15 years. Most of the retained primary teeth required extraction, but no MRONJ occurred. Facial photographic assessment revealed that FD increased and became more asymmetric by the age of 19 years. Although the FD recurred after surgery, peaking at the age of 25, the asymmetry improved afterwards. There are many factors that dental professionals should be aware of when treating MAS patients, such as the administration of bisphosphonate preparations, changes in FD over time, and malocclusion. It is important for dental professionals to provide appropriate oral health instruction to MAS patients and to continue long-term oral management to prevent oral disease.

## Figures and Tables

**Table 1:** Landmark and reference lines.

**Point**	
A	Right endocanthion
B	Left endocanthion
C	Midpoint of bilateral endocanthion
D	Subnasale
E	Intersection of lines (II) and (III)
F	Intersection of line (II) and the right side of the face
G	Intersection of line (II) and the left side of the face
Line	
(I)	Line joining bilateral inner canthi
(II)	Line parallel to line (I) that passes through D
(III)	Line perpendicular to line (I) that passes through C

**Table 2:** Evaluation items for asymmetry.

Ratio	Formula (%)
Asymmetry ratio from midline	|(EF − EG)/(EF + EG)| × 100
Asymmetry ratio from subnasale	|(DF − DG)/(DF + DG)| × 100

**Table 3:** Changes in fibrous dysplasia.

Age	Asymmetry Ratio from Midline (%)	Asymmetry Ratio from Subnasale (%)
14 y 9 m	0.94	4.46
17 y 3 m	7.19	12.66
19 y 9 m (Before osteotomy)	15.36	25.89
19 y 10 m (After osteotomy)	0.78	9.54
20 y 9 m	1.88	6.82
21 y 9 m	1.01	2.61
23 y 3 m	2.11	7.91
25 y 1 m	9.11	20.85
27 y 4 m	6.85	18.38
30 y 5 m	4.86	14.38

**Table 4:** Timeline of patient.

Age	Occurrences
5 y	Swelling in the left cheek region
9 y	Diagnosis of MAS and precocious puberty, and start of bisphosphonate therapy
11 y	Optic canal decompression surgery
13 y 7 m	First visit to our department
15 y 6 m	Left maxillary primary first molar extraction
19 y 5 m	Discontinuation of bisphosphonate therapy
19 y 9 m	Maxillary and mandibular osteotomies
24 y 10 m	Left mandibular primary second molar extraction
30 y 3 m	Left mandibular primary canine extraction

## Data Availability

Data are contained within this article.
